# Efficiency scale for scattering luminescent particles linked to fundamental and measurable spectroscopic properties

**DOI:** 10.1038/s41598-023-32933-6

**Published:** 2023-04-17

**Authors:** Christian Würth, Thomas Behnke, Jonas Gienger, Ute Resch-Genger

**Affiliations:** 1 Division Biophotonics, Bundesanstalt für Materialforschung und –prüfung (BAM), Richard-Willstaetter Str. 11, 12489 Berlin, Germany; 2 Physikalisch-Technische Bundesanstalt (PTB), Abbestr. 2–12, 10587 Berlin, Germany

**Keywords:** Techniques and instrumentation, Theory and computation, Optical physics, Techniques and instrumentation, Optical spectroscopy, Fluorescence spectroscopy, Colloids, Fluorescent probes

## Abstract

Comparing the performance of molecular and nanoscale luminophores and luminescent micro- and nanoparticles and estimating achievable signal amplitudes and limits of detection requires a standardizable intensity scale. This initiated the development of the relative MESF (number of molecules of equivalent soluble fluorochromes) and ERF (equivalent reference fluorophores) scales for flow cytometry and fluorescence microscopy. Both intensity scales rely on fluorescence intensity values assigned to fluorescent calibration beads by an intensity comparison to spectrally closely matching fluorophore solutions of known concentration using a spectrofluorometer. Alternatively, the luminophore or bead brightness (*B*) can be determined that equals the product of the absorption cross section (*σ*_a_) at the excitation wavelength (*σ*_a_(λ_ex_)) and the photoluminescence quantum yield (*Φ*_pl_). Thereby, an absolute scale based on fundamental and measurable spectroscopic properties can be realized which is independent of particle size, material, and luminophore staining or labeling density and considers the sensitivity of the optical properties of luminophores to their environment. Aiming for establishing such a brightness scale for light-scattering dispersions of luminescent particles with sizes exceeding a few ten nanometers, we demonstrate how the brightness of quasi-monodisperse 25 nm, 100 nm, and 1 µm sized polystyrene particles (PSP), loaded with two different dyes in varying concentrations, can be obtained with a single custom-designed integrating sphere setup that enables the absolute determination of *Φ*_pl_ and transmittance and diffuse reflectance measurements. The resulting *Φ*_pl_, *σ*_a_(λ_ex_), imaginary parts of the refractive index, and calculated *B* values of these samples are given in dependence of the number of incorporated dye molecule per particle. Finally, a unitless luminescence efficiency (*LE*) is defined allowing for the direct comparison of luminescence efficiencies of particles with different sizes.

## Introduction

In the last decades, nanoparticles (NPs) and microparticles (MPs), stained or encoded with different types of molecular and nanocrystalline luminophores, have been increasingly used in the life and material sciences. Typical applications range from optical reporters for fluorescence assays and bioimaging and drug delivery systems over printable authentication tags and bead-based platforms for flow cytometry, fluorescence microscopy, and immune-separation to particle sensors and calibration tools for different fluorescence methods, particularly for flow cytometry^1–13^. Most fluorescence methods exploiting emissive NPs and MPs like fluorescence spectroscopy, microfluorometry, fluorescence microscopy, and flow cytometry measure only instrument-specific relative fluorescence intensities^14^. The reliable comparison of fluorescence measurements between different instruments and different laboratories requires an instrument calibration to determine and consider instrument-specific signal contributions such as the wavelength-dependent spectral responsivity of the instrument´s detection channel, that affect the measured emission spectra^15,16^. For the quantification of, e.g., analytes or the comparison of different fluorescent samples with different fluorescence techniques, commonly a relative calibration of the fluorescence intensity scale is performed using fluorophore solutions of known concentrations, luminescence properties, and particularly emission spectra closely matching that of the sample utilizing the same instrument settings as applied for the measurement of the sample^17^. This is straightforward for transparent luminescent samples, e.g., sensing applications or the quantification of luminophores with chromatographic separation techniques like high performance liquid chromatography (HPLC) with fluorescence detection, yet it is challenging for light-scattering systems. However, most dispersions of broadly used fluorescent NPs and MPs scatter the excitation light, depending on their size and the refractive index and environment of the particles. This can affect their fluorometric characterization and particularly measurements of their absorption features with common spectrophotometers and spectrofluorometers designed for the measurement of transparent samples.

The importance of quantifying scattering luminescent objects for example for flow cytometry used for blood, cell, and biomarker analysis in strongly regulated areas like the health sector and medical diagnostics recognized 30 years ago called for the establishment of a comparable and standardizable fluorescence intensity scale^18^. This initiated the development of the so-called relative MESF (number of molecules of equivalent soluble fluorochromes)^19,20^ and ERF (equivalent reference fluorophores)^11^ scales for measuring and quantifying the fluorescence intensity of fluorescent objectives in flow cytometry with the aid of luminescent calibration beads^21^. This concept is also utilized in fluorescence microscopy. The MESF and ERF scale rely on the comparison of the integral fluorescence intensities of a dispersion of dye-stained particles with a narrow size distribution of known concentration with a solution of a molecular fluorophore of known concentration with a closely matching emission spectrum. Thereby, the fluorescence intensity of the beads is expressed in terms of the intensity of this molecular fluorophore and the quantitative determination of the spectroscopic key parameters absorption cross section (*σ*_a_(λ)) and photoluminescence or fluorescence quantum yield (*Φ*_pl_) of the dye-stained beads is circumvented. These relative intensity calibration concepts have meanwhile led to the broad availability of a multitude of calibration beads with assigned MESF or ERF values adapted to the optical properties of typical fluorescent labels applied in flow cytometry. In addition, the reliability and accuracy of these concepts have been assessed for different types of core-stained or fluorophore labeled calibration beads by several interlaboratory comparisons of expert groups and different flow cytometers in the last decades. Thereby, limitations, caused, e.g., by the microenvironment- and dye-concentration dependent fluorescence efficiency of most fluorophores and different instrument designs including optical components used for flow cytometers have been examined^22–26^.

As an alternative to a relative fluorescence intensity scale, an absolute scale based on the fundamental spectroscopic properties *σ*_a_(λ_ex_) and *Φ*_pl_^21,27^, the ratio of the number of emitted photons per number of absorbed photons^28^, can be realized and used for quantification, e.g. in imaging studies^29^. The product of these two quantities is termed brightness (*B*) and controls the size of each fluorometrically detected signal from the sample side. This concept has been suggested for single molecules^30^, yet not for light-scattering dispersions of luminescent particles as light scattering renders the determination of *σ*_a_(λ_ex_) and *Φ*_pl_ more difficult and requires special instrumentation. *σ*_a_(λ_ex_) of particle dispersions can be derived from radiation transport theory, utilizing a spectrophotometer equipped with an integrating sphere for the measurement of the wavelength-dependent transmittance and diffuse reflectance (*R*_d_). *Φ*_pl_ of light-scattering luminescent samples can be optically only accurately determined by integrating sphere spectroscopy^27,31^ and the increasing interest in such measurements initiated a renaissance of integrating sphere spectroscopy in the last decade^32–37^. For particles with sizes below 100 nm, where scattering is more or less isotropic, there are also very few reports on alternative relative spectroscopic methods, that are, however, more tedious and error prone. This includes a subtracting-based scattering correction of the measured absorbance at the excitation wavelength^38^, the addition of scatterers to the fluorescence quantum yield standard employed for the relative determination of *Φ*_pl_, thereby matching the scattering features of sample and standard^39^, and the determination of the complex dielectric function of dye-loaded particles and consideration of the influence of Mie scattering^40^.

With the aim to establish a *B* scale for the characterization of the signal relevant properties of luminescent beads, we show in the following how *B* of light-scattering dispersions of luminescent particles can be obtained with a single custom-designed and traceably calibrated integrating sphere setup. This unique setup enables both the absolute determination of *Φ*_pl_ values as well as measurements of *R*_d_, collimated transmittance (*T*_c_), and diffuse transmission (*T*_d_) spectra of particle dispersions^27^. The determination of *B* values is demonstrated for dispersions of monodisperse 100 nm and 1 µm sized polystyrene particles (PSP), loaded with different concentrations of Nile Red and Itrybe molecules, chosen to vary in their solvatochromic behavior and spectral overlap between their absorption and emission bands (Stokes shift) and hence, concentration-dependent reabsorption^41^. Nile Red is a frequently employed polarity probe^42,43^ and polystyrene particles loaded with Itrybe and Nile Red were used by us before for dye loading studies^41,42^ and as tumor specific NIR-probe^44^ or the lifetime-based encoding of cells^45^. In this publication, we reevaluate previously determined *B* values of such dye-stained beads^42^ and provide the imaginary part of the refractive index. In addition, *B* and efficiency values from measured *σ*_a_(λ_ex_) and *Φ*_pl_ data are given in dependence of the number of incorporated dye molecule per particle.

## Materials and methods

The experimental procedures used for the dye staining of the differently sized carboxyl-functionalized PSP and the absolute determination of *Φ*_pl_ values with our custom-designed integrating sphere setup enabling measurements of the fluorescence, *R*_d_, *T*_c_, and *T*_d_ spectra of particle suspensions have been partly previously reported^27,31,41,42,44,46^.

### Materials

Carboxyl-functionalized PSP with sizes of 25 nm, 100 nm, and 1 µm were purchased from Kisker Biotech GmbH. All particles were ultrasonically treated prior to use. Nile Red and Itrybe were obtained from Fluka GmbH and Otava Ltd, respectively. The solvents tetrahydrofuran (THF), dibutylether (BOB), and ethanol (EtOH) were of spectroscopic grade and purchased from Merck. All dyes and solvents were used as received.

### Instrumentation

For spectrally resolved measurements in the wavelength region from about 350 to 1000 nm, a custom designed and traceably calibrated integrating sphere setup was used enabling the free choice of the excitation wavelength^27^. This previously reported setup^31,46^ consisted of a 450 W xenon lamp, coupled to a single monochromator with 1200 L/mm gratings blazed at 330 nm, 500 nm, and 630 nm and an integrating sphere with a diameter of about 15 cm, coated with Spectraflect (Labsphere GmbH; sphere reflectivity of about 97% in the vis/NIR) as the sample compartment. The integrating sphere was coupled with a quartz fiber bundle to an imaging spectrograph (Shamrock 303i, Andor Technology PLC), equipped with 600 L/mm gratings, blazed at 500 nm and 750 nm that was attached to a Peltier cooled (183 K) thinned back side illuminated deep depletion charge coupled device (CCD array; 1024 × 256 pixel). The optical fiber was shielded from direct reflexes with several baffles to guarantee the detection of only diffuse radiation. To account for its fluctuations and for the measurements of the collimated transmittance two Peltier-cooled custom-designed silicon trap detectors^47^ were implemented into the integrating sphere setup.

To realize multiple measurement configurations or illumination conditions for the spectrally resolved measurements of the emission, reflection, and transmittance and an indirect sample illumination, the integrating sphere was equipped with several ports for the coupling of light into the sphere and sample mounting. For the absolute determination of *Φ*_pl_, the sample was center-mounted inside the integrating sphere using a custom-designed Spectraflect-coated cuvette holder. The excitation light was focused with two off-axis parabolic mirrors into the middle (sample position) of the integrating sphere, thereby imaging the exit slit of the excitation monochromator onto the middle of the cuvette (see Supplementary Fig. S1)^31^. The radiant power reaching the sample was adjusted with an aperture, located in front of the integrating sphere. For the precise positioning of the cells inside the integrating sphere, a small HeNe laser was used in addition to the cuvette holder. The laser light reflected from the glass/air interface of the cuvette window was measured outside the integrating sphere.

For transmission and reflection measurements, a collimated beam is required. Therefore, the integrating sphere setup was slightly modified. The parabolic off-axis mirror 2 (see Supplementary Information (SI), Fig. S1) was exchanged by a plane mirror. Thereby, the spot size for reflection and transmission measurements could be adjusted with apertures behind the opened exit slit of the excitation monochromator. Custom designed sample holders for thin films and small volume cells were placed in front or behind the integrating sphere. The cells were designed to exactly fit the ports of the integrating sphere. Typical sample thickness for transmission and reflection measurements ranged from 200 µm to 1 mm with a diameter of 2.54 cm.

### Absorption measurements of transparent samples

The absorption spectra of transparent solutions of the dyes Nile Red and the dyes released from the dye-stained PSP upon addition of THF were measured with Cary5000 UV–Vis-NIR spectrophotometer using 1 cm quartz cells from Hellma.

### Dynamic light scattering (DLS) measurements

Dynamic light scattering measurements for particle size determination were performed with the Zetasizer from Malvern.

## Methods

### Preparation of dye-stained polystyrene particles

Commercial carboxylated PSP were stained with Nile Red and Itrybe via a previously reported swelling procedure^27,41,42^. In short, Nile Red and Itrybe were dissolved in THF in concentrations from 0.1 to 5 mmol/L. 100 µL of the dye-containing solution were added to 600 µL of an aqueous PSP suspension (3 mg/mL). 25 nm PSP were incubated for 30 min, whereas 100 nm and 1 µm PSP were incubated for 1 h. Then, 800 µL of water were added and the suspension was centrifuged (1 µm PSP (5,000 g, 10 min) and 100 nm PSP (15,000 g, 40 min) in a Eppendorf centrifuge 5415D, 25 nm PSP (75,000 g, 45 min) in a Beckman Coulter centrifuge Avanti J-20 XP. The stained PSP were washed twice and resuspended in bidistilled water.

### Determination of the average dye loading density

The number of incorporated dye molecules per particle was photometrically determined after dissolving the particles by addition of THF^48^. In short, the stained PSPs were centrifuged, the supernatant removed and dissolved in THF. The dye concentration released from 3 mg PSP was calculated from absorbance measurements and the molar absorption coefficient, determined in THF in the presence of 0.1 w% dissolved polystyrene. The polystyrene mass concentration was determined by drying and weighing 1 mL of the aqueous suspension and the number of PSP per mg was determined by considering the particle volume and PS density (1050 kg/m^3^).

## Method development and theoretical background

### Workflow for particle brightness and efficiency determination

**Figure 1:**
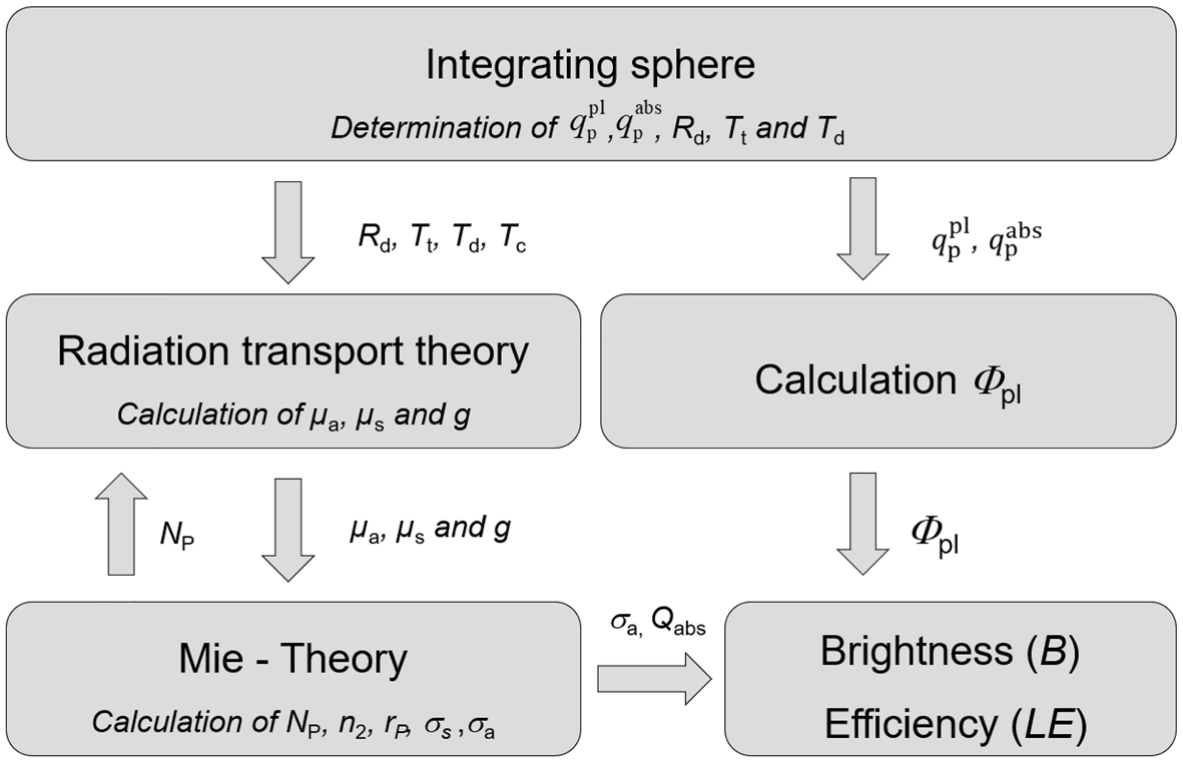
Flow chart of data acquisition and analysis: absorbed (${q}_{\mathrm{p}}^{\mathrm{abs}}$) and emitted photon flux (${q}_{p}^{pl}$). Diffuse reflectance (*R*_d_), total (*T*_t_), diffuse (*T*_d_), and collimated transmittance (*T*_c_), scattering coefficients (*µ*_s_), absorption coefficients (*µ*_a_), scattering anisotropy factor (*g*), Fluorescence quantum yield (*Φ*_pl_), number of particles per unit volume (*N*_P_), particle radius (*r*_P_), imaginary part of the refractive index (*n*_2_), absorption cross-section (*σ*_a_), absorption efficiency (*Q*_abs_), and the particle luminescence efficiency (*LE*).

Our procedure of data acquisition for particle brightness and efficiency determination based on integrating sphere spectroscopy follows the schematic presentation illustrated in Fig. 1. For the determination of *Φ*_pl_ values, the absorbed (${q}_{\mathrm{p}}^{\mathrm{abs}}$) and emitted photon flux (${q}_{p}^{pl}$) are determined (see section on the “Determination of photoluminescence quantum yields”). In a next step reflection and transmission measurements were performed (see section on “Reflection and transmission measurements”) yielding *R*_d_, *T*_t_, *T*_d_ and *T*_c_. With these experimentally determined parameters, *Φ*_pl_ can be directly calculated (see section on the “Determination of photoluminescence quantum yields”). By solving the radiation transport theory, the scattering (*µ*_s_), absorption coefficients (*µ*_a_) and scattering anisotropy factor (*g*) can be calculated (see section on the calculation of µ_a_, µ_s_, and *g*). By implementing Mie theory, also the number of particles per unit volume (*N*_P_) the particle radius (*r*_P_) and the imaginary part of the refractive index (*n*_2_) (section on the “Determination of particle properties”) are determined. The resulting *σ*_a_(λ_ex_) and *Φ*_pl_ values are finally used for the calculation of *B* and the luminescence efficiency (*LE*)***.***

### Optical response of an integrating sphere

The optical response of an integrating sphere is dependent on its configuration (sample holder, baffle, port sizes etc.). For an ideal sphere, the magnification factor *M*(λ) is given by^49^:

1 $$M(\lambda )=\frac{{\rho }_{0}(\lambda )}{1-{\rho }_{S}(\lambda )(1-{\sum }_{i=0}^{n}{f}_{i})-{\sum }_{i=0}^{n}{\rho }_{i}(\lambda ){f}_{i}}=\frac{{\rho }_{0}(\lambda )}{1-\overline{\rho (\lambda )}}$$

**Figure 2:**
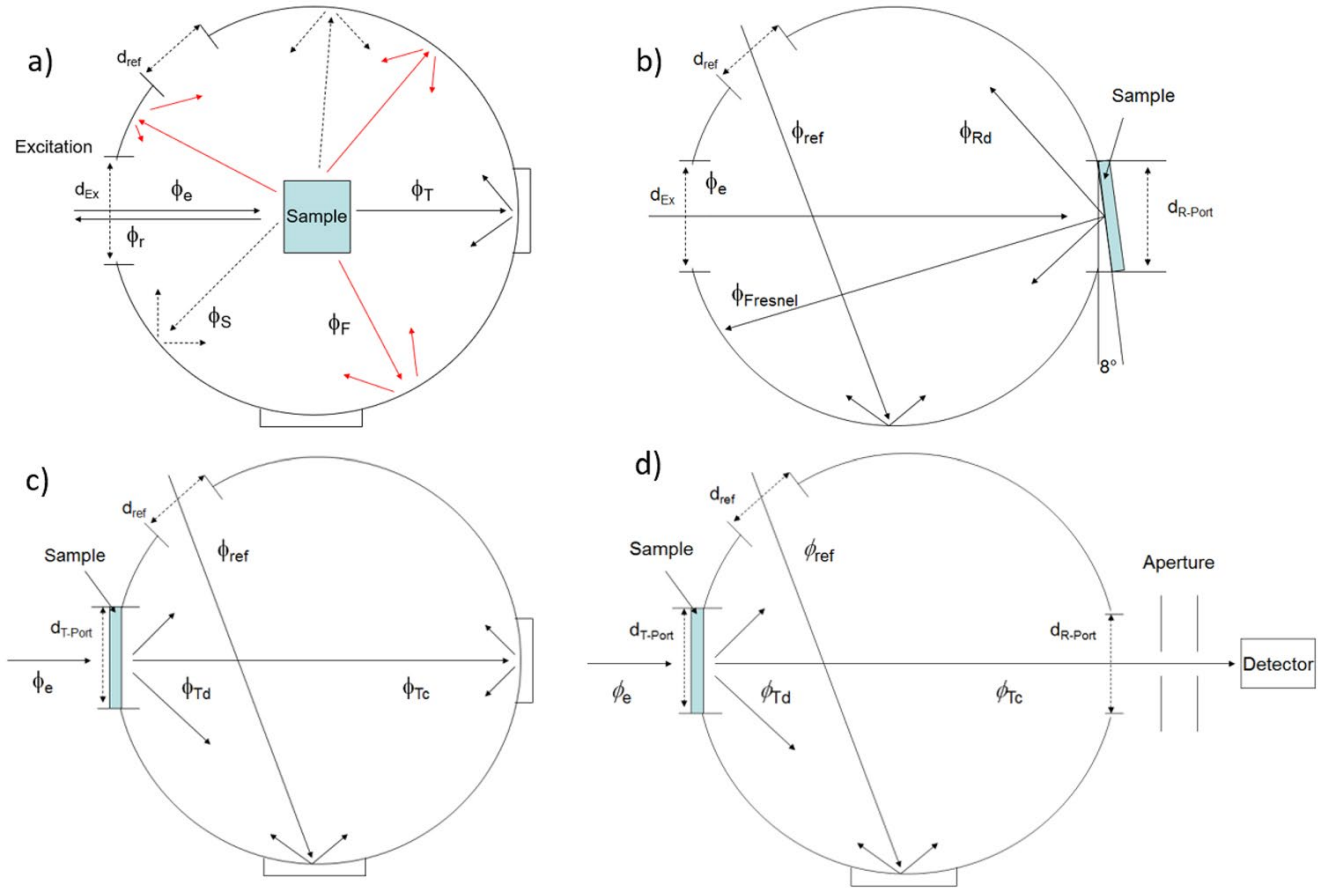
Configurations of the integrating sphere setup, see also Supplementary Fig. S1, and the corresponding light fluxes (eq. S3) employed for (**a**) ${\Phi }_{pl}$ measurements, (**b**) *R*_d_ measurements, and (**c, d**) the measurement of *T*_t_, *T*_d_, and *T*_c_.

$\overline{\rho } (\lambda )$ represents the average wavelength-dependent reflectivity of the sphere surface which depends on the port areas *A*_*i*_ with their corresponding reflectivities $\rho_{i} (\lambda )$ and the sphere’s surface area *A*_S_ and reflectivity *ρ*_S_(λ), respectively, (*f*_i_ = *A*_i_/*A*_S_). The incident radiant flux *ϕ*_e_ is diffusely reflected from a surface area with a reflectance *ρ*_0_(λ) (see Eq. 1, Fig. 2). The resulting the radiant flux *ϕ*_S_ reaching the sphere surface and generating the detector signal *I* is dependent on *ϕ*_e_ and *M*(λ):

2 $$\Phi_{S} = \Phi_{e} M\left( \lambda \right).$$

By further taking into account the detector surface *A*_D_ and the spectral responsivity of the detection system *s*(λ), *I *can be written as:

3 $$I = s\left( \lambda \right)\Phi_{s} \frac{{A_{D} }}{{A_{S} }}.$$

Thus, each change of the integrating sphere configuration (see also Fig. 2) or surface, e.g., the exchange of a sample and a reference, changes the response of the integrating sphere setup and must be considered.

### ***Determination of photoluminescence quantum yields***${(\Phi }_{pl})$

The photoluminescence quantum yield is defined as the quotient of the number of absorbed photons (*N*_abs_) and the number of emitted photons (*N*_em_) by a sample^27,42,46,50^, see Eq. (4).

4 $${\Phi }_{pl}=\frac{{N}_{em}}{{N}_{abs}}$$

${\Phi }_{pl}$ of scattering samples like the suspensions of our dye-stained PSP can be only reliably determined by integrating sphere spectroscopy^51^. This involves the determination of the absorbed photon flux (${q}_{p}^{abs}({\lambda }_{\text{ex}})$) and the emitted photon flux (${q}_{p}^{em}({\lambda }_{\text{em}})$) of the sample (see Eq. (5) and Fig. 2a))^31,46^ with an integrating sphere setup.

5 $$\Phi_{pl} = \frac{{\mathop \smallint \nolimits_{{\lambda_{{{\text{em}}_{1} }} }}^{{\lambda_{{{\text{em}}_{2} }} }} \frac{{\left( {I_{x} \left( {\lambda_{{{\text{em}}}} } \right) - I_{b} \left( {\lambda_{{{\text{em}}}} } \right)} \right)}}{{s\left( {\lambda_{{{\text{em}}}} } \right)}}\lambda_{{{\text{em}}}} d\lambda_{{{\text{em}}}} }}{{\mathop \smallint \nolimits_{{\lambda_{ex} - \Delta \lambda }}^{{\lambda_{ex} + \Delta \lambda }} \frac{{\left( {I_{b} \left( {\lambda_{{{\text{ex}}}} } \right) - I_{x} \left( {\lambda_{ex} } \right)} \right)}}{{s\left( {\lambda_{ex} } \right)}}\lambda_{{{\text{ex}}}} d\lambda_{{{\text{ex}}}} }} = \frac{{q_{p}^{{{\text{em}}}} }}{{q_{p}^{{{\text{abs}}}} }}$$

The emitted photon flux follows from the blank and spectrally corrected emission spectrum of the sample (*I*_x_(*λ*_em_)) integrated over the emission band. The absorbed photon flux is calculated from the difference between the transmitted excitation light obtained with the blank (*I*_b_(*λ*_ex_)) and the sample (*I*_x_(*λ*_ex_)), within the wavelength region of the excitation bandwidth Δ*λ.*

### Reflection and transmission measurements

Reflection and transmission measurements were performed with the same integrating sphere setup as the quantum yield measurements. The sample was filled in cylindrical small-volume quartz cells with a thickness of 1 mm. For every recorded measured signal of a standard (index std) or sample (index S) or empty sphere (index empty) a reference measurement was done to account for changes of the magnification factor *M*(λ). For the calculation of the reflectance and transmittance the spectrally resolved signals *I* determined the integrating sphere were integrated in the spectral range of the excitation light $E = \int\limits_{{\lambda_{ex} - \Delta \lambda }}^{{\lambda_{ex} + \Delta \lambda }} {I(\lambda_{{{\text{ex}}}} )} d\lambda_{{{\text{ex}}}}$. The spectrally resolved detection has the advantage that also strongly fluorescent samples can be investigated. Integrating spheres equipped only with photomultiplier tubes or photodiodes cannot distinguish between excitation/incident light and the red shifted luminescence and are therefore not suited for reflectance and transmission of luminescent materials.

### Diffuse reflectance (R_d_)

For measurements of *R*_d_ the sample was mounted with special sample holders on the back of the integrating sphere (R-Port Fig. 2b)), *R*_d_ was then calculated with respect to NIST certified diffuse reflectance standards according to:

6 $${R}_{d}(\lambda )={R}_{\text{Std}}(\lambda ){\left(\frac{{E}_{e}(\lambda )-{E}_{D}(\lambda )}{{E}_{\text{Ref}}(\lambda )-{E}_{D}(\lambda )}\right)}_{S}{\left(\frac{{E}_{\text{Ref}}(\lambda )-{E}_{D}(\lambda )}{{E}_{e}(\lambda )-{E}_{D}(\lambda )}\right)}_{\text{Std}}$$

*R*_Std_(λ) equals the certified *R*_d_ value of the reflectance standard, *E*_e_(λ) and *E*_Ref_(λ) stand for the detector signal with direct and indirect sample illumination, respectively. *E*_*D*_ indicates a dark measurement to remove a signal offset. The subscripts S and Std stand for measurements of the sample and the standard mounted at the reflectance port, respectively. The first reflection from the sample’s glass surface (*R*_Fresnel_) was included in the measurements.

### Transmittance (T)

For each transmission measurement, the sample was mounted at the entrance port of the integrating sphere. The transmitted radiation (*T*_total_) can be separated into two parts, the directly or collimated (*T*_c_) and the diffusely (*T*_d_) transmitted radiation:

7 $$T_{{{\text{total}}}} (\lambda )\, = \,T_{{\text{d}}} (\lambda )\, + \,T_{{\text{c}}} (\lambda )$$

Photons, that are collimatedly transmitted, pass the sample without interaction and have thus the same propagation direction as the incident photons, while diffusely transmitted photons are scattered at least once. The total transmittance was measured with the integrating sphere configuration shown in in Fig. 2c) and calculated with Eq. (8). With the same configuration but an open port on the back of the integrating sphere (R-port), the diffuse transmittance can be determined, see Fig. 2d) and Eq. (8).

8 $${{T}_{T,D}}(\lambda )={\left(\frac{{E}_{e}(\lambda )-{E}_{D}(\lambda )}{{E}_{\mathit{Re}f}(\lambda )-{E}_{D}(\lambda )}\right)}_{S}{\left(\frac{{E}_{\mathit{Re}f}(\lambda )-{E}_{D}(\lambda )}{{E}_{e}(\lambda )-{E}_{D}(\lambda )}\right)}_{Empty}$$

In principle *T*_c_ can be calculated from the total and diffuse transmitted photon flux (Eq. 7). But the open R-port for measurements of *R*_d_ causes losses of diffusely scattered photons leading to an incorrect calculation of *T*_c_. These errors correlate with the size of the R-port and the diameter of the integrating sphere. Therefore, a trap detector was used to measure *T*_c_. Our trap detector consists of three silicon photodiodes arranged in a 60° geometry to trap the incident light. This trap detector with an aperture of 0.5 mm was positioned in a distance of 1.5 m behind the sample to reduce the solid angle for scattered and emitted photons contributing to measurements of *T*_c_. *T*_c_ was then calculated from the signals of the trap detector *I*_Trap_:

9 $${T}_{c}(\lambda )=\frac{{I}_{Trap}^{sample}(\lambda )-{I}_{Trap}^{Dark}(\lambda )}{{I}_{Trap}^{empty}(\lambda )-{I}_{Trap}^{Dark}(\lambda )}$$

### Radiation transport

In strongly scattering media the propagation of light can be described by the radiation transport theory:

10 $$\begin{aligned} \frac{{dL\left( {r,s,\lambda } \right)}}{ds} & = \;\; - \left( {\mu_{a} \left( \lambda \right) + \mu_{s} \left( \lambda \right)} \right)\;L\left( {r,s,\lambda } \right)\;\; \\ & \;\;\; + \;\;\mu_{s} \mathop \smallint \limits_{4\pi } p\left( {s,\tilde{s},\lambda } \right)\;L\left( {r,\tilde{s},\lambda } \right)\;\;d\Omega \;\; + S\left( {r,s,\lambda ,\lambda_{em} } \right) \\ \end{aligned}$$

The light distribution inside a sample is defined by *L*(r,s), which stands for the vectored radiance at the point r in the propagation direction *s* (illustrated in the SI in Fig. S2). To facilitate reading we neglect vector notations here. *L*(*r,s*) depends on the number of absorbing and scattering particles in the sample described by *µ*_a_ and *µ*_s,_ the absorption and scattering coefficients. *p*(*s,* $\tilde{s}$ λ) is the phase-function for scattering processes and $d\Omega$ is the solid angle. *S*(*r,s*) is a source term for the isotropically emitted fluorescence in the sample at the point *r* and depends on the density of the excited fluorophores and *L*(*r,s*) at the fluorophore position. The propagation of light is illustrated in the SI in Fig. S2. With the knowledge of the three parameters *T*_t_, *T*_c_ (or *T*_d_), and *R*_d_, the radiation transport theory can be solved with approaches like the Kubelka–Munk theory, Monte Carlo methods, and the inverse adding doubling (iAD) method to calculate the sample-specific parameters µ_a_, µ_s_, and *g*^52–56^. g is the mean cosine of the scattering phase function, which can be calculated from Mie theory. Typically, the phase function is reduced to a more simplified model like the Henyey-Greenstein phase function (HG) or the Gegenbauer Kernel phase function^57,58^. To solve the radiation transport theory and determine the wavelength-dependent parameters *µ*_a_(λ), *µ*_s_(λ), and *g*(λ), we used the freely accessible iAD method by Prahl et al.^52^. Here the HG phase function is already implemented, which best fits the scattering phase function of polystyrene particles. For luminescent samples, the source term *S*(r,s) which depends on *σ*_a_(λ_ex_) and *Φ*_pl_ of the luminophore has to be eliminated. Also, the first reflection of the incident radiation has to be considered.

With our approach, we detect the spectrally resolved transmitted and reflected photon fluxes (see section on “Reflection and transmission measurements”). Therefore, we could separate emitted photons from the incident/transmitted light and calculate the undisturbed reflection and transmittance. This allows the solution of Eq. (9) without taking into account the source term for the fluorescence (*S*(r,s)).

### Determination of particle properties

The scattering and absorption coefficients determined from reflection and transmission measurements and the solution of the radiation transport theory are effective sample parameters and do not directly reveal information about single particle properties. If the number of particles per unit volume (*N*_p_) is known the absorption and scattering cross sections (${\upsigma }_{\mathrm{a},\mathrm{s}}(\uplambda ))$ can be directly calculated from the absorption coefficient ${\mu }_{a}(\lambda )$ calculated via the iAD method.

11 $${\sigma }_{a,s}(\lambda )=\frac{{\mu }_{a,s}(\lambda )}{{N}_{P}}$$

For polydisperse samples, this cross section is the average over the size distribution. Further, if also the particle radius *r*_p_ is known the scattering and absorbance efficiencies (*Q*_a,s_ $(\lambda )$) can be determined. *Q*_a,s_ $(\lambda )$ is defined in Eq. (12) as the ratio of the corresponding cross section and the physical cross section of a particle.

12 $${Q}_{a,s}(\lambda )=\frac{{\sigma }_{a,s}(\lambda )}{\pi {r}_{p}^{2}}$$

The determination of *N*_p_ can be rather challenging. With the knowledge of the *r*_p_ and its distribution a defined sample amount can be dried, and the number of particles can be calculated from the mass resulting and density of the particle material. For this method, a sufficiently large sample amount is required. Unfortunately, the dried particles are often not easily redispersed. Also, a flow cytometer or calibrated nanoparticle tracking analysis (NTA) can be used to determine the particle concentration but for these approaches the samples often need to be strongly diluted. *r*_p_ can also be determined, e.g., by scattering methods like dynamic or static light scattering (DLS, SLS) which analyze the scattered light intensity fluctuation or spatial intensity distribution. Also, e.g., small angle X-ray scattering (SAXS) or NTA^59,60^ or image-based techniques like transmission and scanning electron microscopy (TEM, SEM) can be used. The latter image-based methods, that measure individual particles, can be time consuming as a statistically relevant number of particles have to be measured to accurately determine the size distribution.

### Implementation of Mie theory

A straightforward approach not needing any other instrumentation is the theoretical description of optical particle properties via the rigorous scattering theory (Mie theory), i.e., the solution of the wave equation for spherical boundary conditions. σ_s_(λ), σ_a_(λ), *p*(λ), and *g*(λ) can be theoretically predicted and adapted to the solution of the radiation transport theory.

The cross sections (σ_a,s_) are calculated from the fundamental parameters a_n_ and b_n_:

13 $${a}_{n}=\frac{{\psi }_{n}^{\mathrm{^{\prime}}}\left(y\right){\psi }_{n}\left(x\right)-m{\psi }_{n}(y){\psi }_{n}^{\mathrm{^{\prime}}}(x)}{{\psi }_{n}^{\mathrm{^{\prime}}}\left(y\right){\zeta }_{n}\left(x\right)-m{\psi }_{n}(y){\psi \zeta }_{n}^{\mathrm{^{\prime}}}(x)}$$

14 $${b}_{n}=\frac{{m\psi }_{n}^{\mathrm{^{\prime}}}\left(y\right){\psi }_{n}\left(x\right)-m{\psi }_{n}(y){\psi }_{n}^{\mathrm{^{\prime}}}(x)}{{m\psi }_{n}^{\mathrm{^{\prime}}}\left(y\right){\zeta }_{n}\left(x\right)-m{\psi }_{n}(y){\psi \zeta }_{n}^{\mathrm{^{\prime}}}(x)}$$

with *y* = π*md*/λ_med_ and *x* = π*d*/λ_med_ and the Ricatti-Bessel functions ψ_n_ and ζ_n_ These fundamental parameters depend on the complex relative refractive index *m* between the particle (*n*_p_) and the medium (*n*_med_), the particle radius (*r*_P_), and the wavelength in the outer medium λ_med_ = λ/*n*_med_. We used the codes from Mätzler^61^ and modified them to calculate the wavelength-dependent scattering and absorption coefficients. Finally, we generated a least square fitting algorithm based on the Mie- and radiation transport theory. Input parameters for this routine are ${\mu }_{a}(\lambda )$, ${\mu }_{s}(\lambda )$, ${g}_{s}\left(\lambda \right),$ and the material properties, e.g., the refractive indices of the solvent and the particle^62^. As free parameters for the fitting procedure, not only *N*_p_ and *r*_p_ but also the width of the size distribution function was used to consider the polydispersity of the bead dispersion. The fitting procedure finally provides *r*_p_, *N*_p_, and the imaginary part of the refractive index (*n*_2_) of the particles. Alternatively, the real part of the refractive index can be determined if the particles size is known^63^.

To precisely determine *r*_p_ via fitting a characteristic wavelength dependence of ${\mu }_{s}(\lambda )$ is necessary, exhibiting oscillatory behavior (Mie resonances). This depends on the solvent, particle material, and size as well as the investigated wavelength region. E.g., for polystyrene particles with a diameter in the order of 1 µm and larger, dispersed in water, *r*_p_ and *N*_p_ can be determined. For particles, which do not exhibit a characteristic wavelength dependence of *µ*_s_(λ), the knowledge of the particle size is necessary to determine the number of particles per unit volume. This is similar to previously presented method(s) based on *T*_c_.^64^

### Kramers–Kronig relations for the refractive indices of the dye-stained PSP

The real and imaginary parts of the complex refractive index ${n\left(\lambda \right)={n}_{1}\left(\lambda \right)+{\text{i}}\hspace{0.17em}{n}_{2}\left(\lambda \right)}$ of a material are linked by so-called Kramers–Kronig (*KK*) relations. For example, ${n}_{1}\left(\lambda \right)$ is related to ${n}_{2}\left(\lambda \right)$ by

15 $${n}_{1}\left(\lambda \right)-1=-\frac{2}{\pi }{\text{P}}{\int }_{0}^{\infty }\frac{\lambda }{\Lambda }\frac{\lambda }{{\Lambda }^{2}-{\lambda }^{2}}\hspace{0.17em}{n}_{2}\left(\Lambda \right)\hspace{0.17em}{\text{d}}\Lambda$$

Here, $P\int$ denotes the Cauchy principal value integra and $\lambda$ the wavelength in vacuo. Note that the integral is taken over all wavelengths (or frequencies) from zero to infinity. The real part of the refractive index is influenced by the absorption spectrum at all other wavelengths.

The complex refractive index of a PSP containing a dye at an intra-particle molar concentration ${c}^{\text{dye}}$ is given by

16 $$n\left(\lambda \right)={n}^{\text{matrix}}\left(\lambda \right)+{c}^{\text{dye}}\hspace{0.17em}\left[\widetilde{\alpha }\left(\lambda \right)+{\text{i}}\hspace{0.17em}\widetilde{\gamma }\left(\lambda \right)\right]$$

The increment of the imaginary part is related to the molar extinction coefficient of the dyes Nile Red and Itrybe by

17 $$\widetilde{\upgamma }\left(\uplambda \right)=\frac{\mathrm{ln}10}{4\uppi }\hspace{0.17em}\upvarepsilon \left(\uplambda \right)\hspace{0.17em}\uplambda .$$

The unit for the refractive index increments $\widetilde{\alpha }\left(\lambda \right), \widetilde{\gamma }\left(\lambda \right)$ is that of an inverse molar concentration—L/mol. Assuming, that the matrix (i.e., polystyrene) is quasi-non-absorbing in the wavelength range considered here, we set ${n}_{2}^{\text{matrix}}\left(\lambda \right)=0$. Under the assumption that outside of a wavelength range $\left[{\lambda }_{a},{\lambda }_{b}\right]$ (e.g., the visible range) the absorption of the matrix material polystyrene and the dye is similar, the real part of the refractive index increment can be calculated according to

18 $$\widetilde{\alpha }\left(\lambda \right)=-\frac{2}{\pi }{\text{P}}{\int }_{{\lambda }_{a}}^{{\lambda }_{b}}\frac{\lambda }{\Lambda }\frac{\lambda }{{\Lambda }^{2}-{\lambda }^{2}}\hspace{0.17em}\widetilde{\gamma }\left(\Lambda \right)\hspace{0.17em}{\text{d}}\Lambda .$$

If the spectra outside the range $\left[{\lambda }_{a},{\lambda }_{b}\right]$ do not match, additional constant or normal-dispersion terms with positive or negative signs may occur in $\widetilde{\alpha }\left(\lambda \right)$.

For numerical evaluation, we used measured spectra of the molar extinction coefficient $\varepsilon \left(\lambda \right)$ to obtain $\widetilde{\gamma }\left(\lambda \right)$. The integral in Eq. (18) was then evaluated in a range $\left[{\lambda }_{a},{\lambda }_{b}\right]$ covering the main peak of the spectrum. For NR this was 300–700 nm. The spectra were interpolated to a step size of 0.5 nm and the (KK) integral was numerically evaluated^65^. The wavelength grid spacing of the resulting $\widetilde{\alpha }\left(\lambda \right)$ data was increased (downsampling) to the desired value (e.g., 5 nm) for Mie scattering calculations and comparison with measured spectra.

To obtain the complex refractive index for Mie calculations, we determined the average dye loading concentration per particle by photometric measurements of the absorption spectra of the dye molecules released upon particle dissolution with THF (see section on “Determination of particle properties”) and Eq. (18).

## Experimental results and discussion

**Figure 3:**
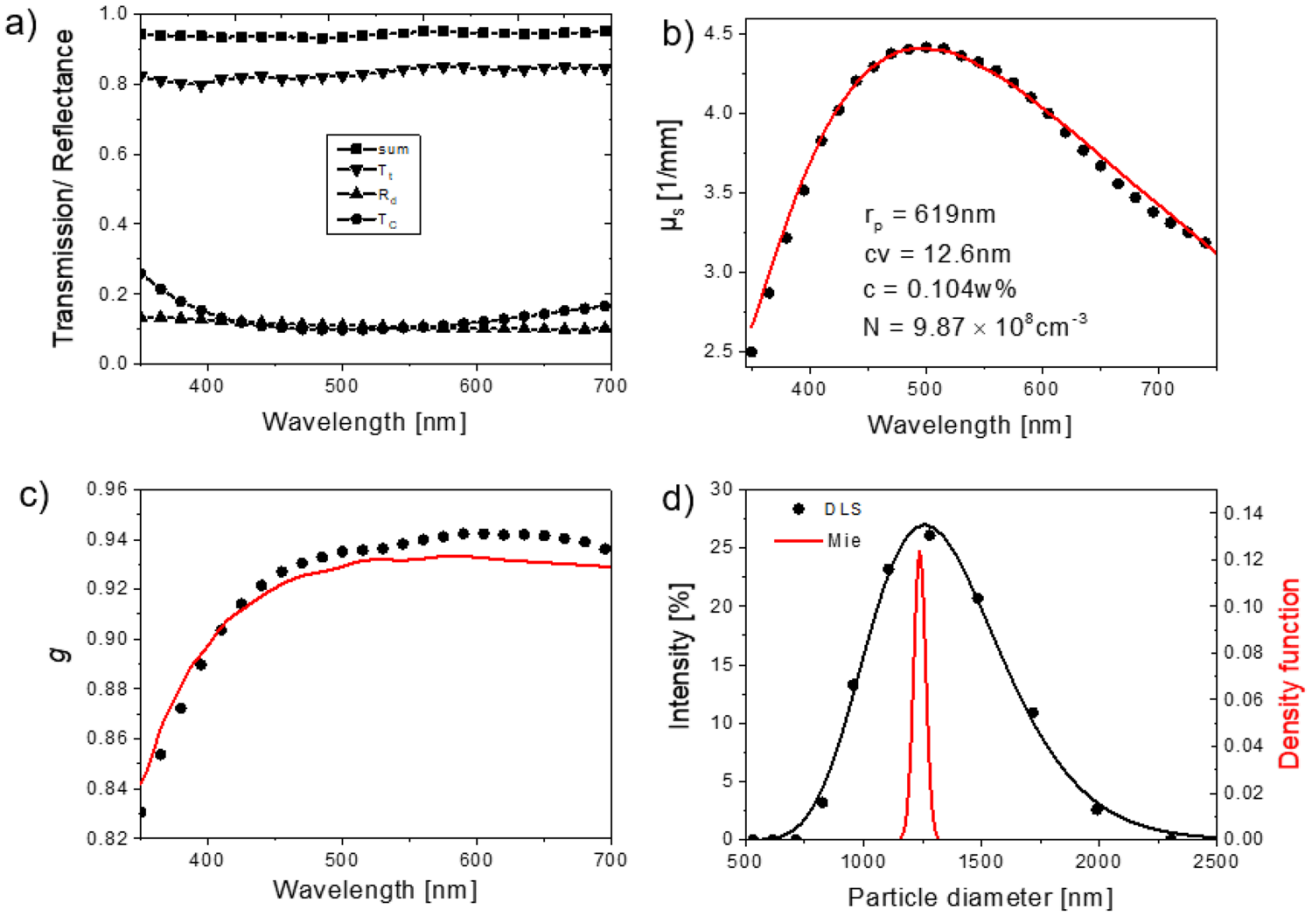
Dispersion of unstained polystyrene particles with about 1.25 µm diameter. (**a**) Diffuse reflectance (*R*_d_, triangles), total (*T*_t_, triangles) and collimated transmittance (*T*_c_, solid circles) and sum of *R*_d_ and *T*_t_ (solid squares). (**b**) Scattering coefficient (*µ*_s_) calculated from the radiation transport theory (solid squares) and Mie theory (red line). (**c**) Anisotropy factor *g* calculated from the radiation transport theory (solid squares) and Mie theory (red line). (**d**) Size distribution function determined with DLS (solid circles, black line) and Mie theory (red line).

To assess our measurement procedures and algorithms, different aqueous dispersions of monodisperse, quasi non-absorbing polystyrene microspheres with varying particle diameters and defined concentrations were used. Examples are shown in Fig. 3 and in the SI in Figs. S3 (*d* ≈ 1 µm) and S4 (*d* ≈ 2.5 µm). Here, we determined *T*_t_, *T*_c_, and *R*_d_ of a dispersion of PSP with a diameter of ca. 1250 nm. The sum of *T*_t_ and *R*_d_ equals 0.95 due to small scattering losses which depend on the sample and the integrating sphere geometry (see Fig. 3a)). These losses can differ for samples with different scattering properties and sample thicknesses but are considered in the iAD routine by Prahl et al.^52^. If not completely compensated these losses result in an offset in the absorption coefficient. Consequently, the radiation transport theory with *T*_t_, *T*_c_, and *R*_d_ as input parameters was solved for all samples to determine the wavelength-dependent scattering coefficient (*µ*_s_(λ), Fig. 3b)) and the wavelength-dependent anisotropy factor (*g*(λ), Fig. 3c)). These results were then fitted with Mie theory to determine *N*_p_ and *r*_p_, the latter was additionally validated with DLS measurements (Fig. 3d yielding *r*_p,DLS_ = 1291 nm. The fitting procedure yields a good agreement between the solution of radiation transport and Mie theory and the resulting particle radius and particle concentrations confirm the suitability of our approach.

**Figure 4:**
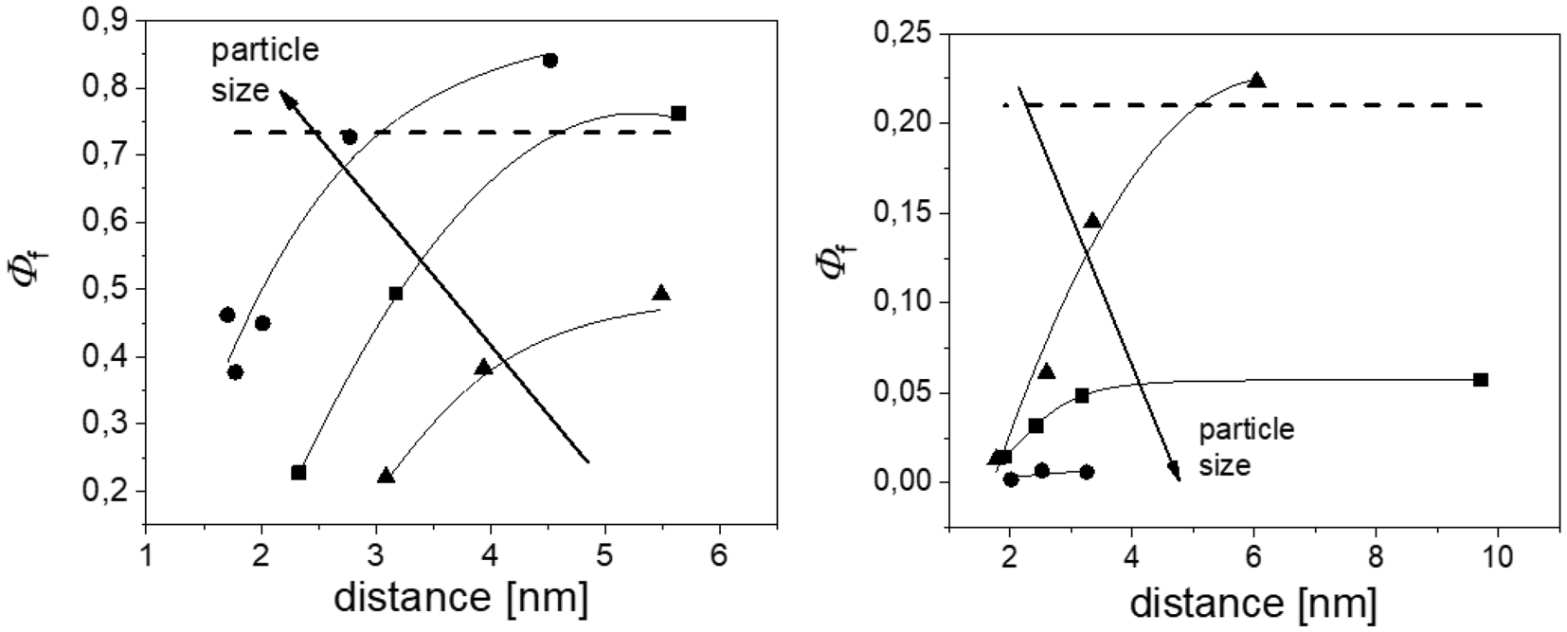
Photoluminescence quantum yields of 1 µm (circles), 100 nm (squares), and 25 nm (triangles) PSP loaded with Nile Red^41^ (left) and Itrybe (right) in dependence on the mean distance between the dye molecules. The lines are only a guide to the eye. The dashed lines indicate the max. ${\Phi }_{\mathrm{pl}}$ in solution of the free dyes, i.e., Itrybe dissolved in ethanol and Nile Red in BOB.

**Figure 5:**
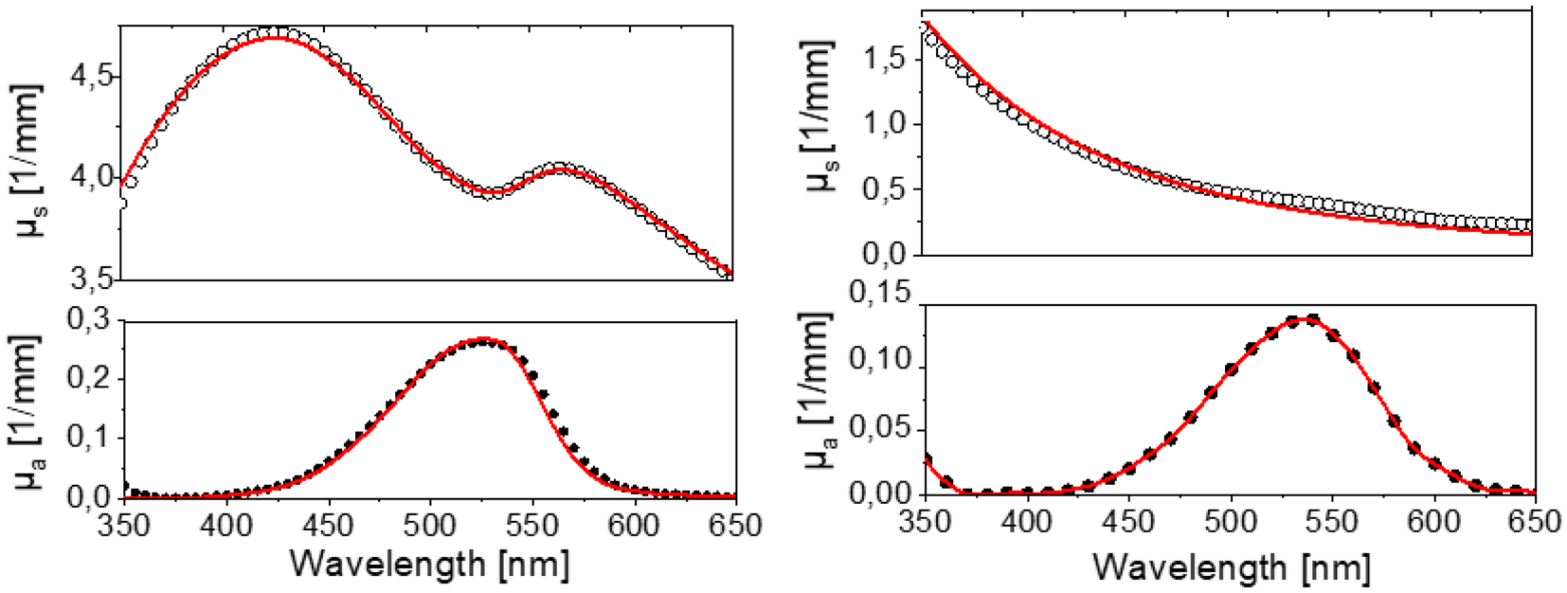
Example of calculated scattering coefficients (µ_s_, top, open circles) and absorption coefficients (µ_a_, bottom, filled circles) from reflection and transmittance measurements of Nile Red-loaded 1 µm (left) and 100 nm (right) PSP. Fits of Mie theory are indicated by red lines.

**Figure 6:**
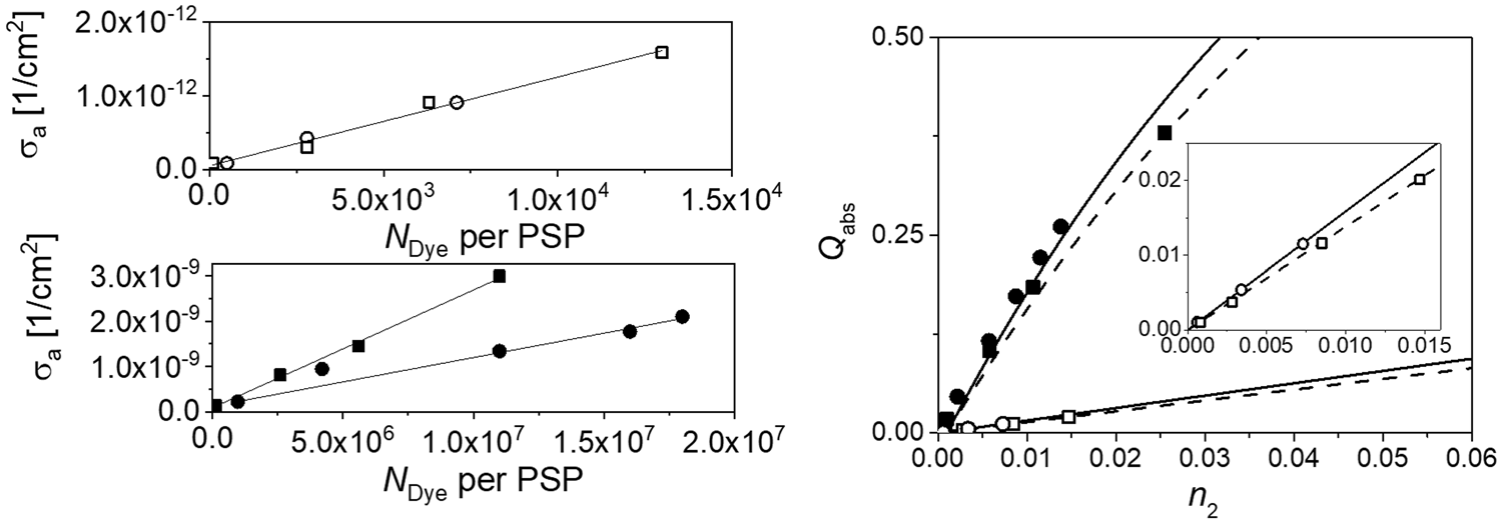
Left: Absorption cross sections of 100 nm (top) and 1 µm (bottom) PSP loaded with Nile Red (circles) and Itrybe (squares) in dependence of the incorporated number of dye molecules per particle. Right: Absorption efficiency (*Q*_abs_) of the dye-loaded PSP in dependence of the imaginary part for the refractive index *n*_2_ for 1 µm PSP (solid symbols) and 100 nm PSP (open symbols) loaded with Nile Red (circles) and Itrybe (squares).

To evaluate our method with absorbing and luminescent particles, quasi-monodisperse model systems were prepared and characterized as described in the “Methods” section. We focussed on particles with sizes of 25 nm, 100 nm, and 1 µm, the latter exhibiting strong anisotropic scattering behaviors (*g* > 0). The normalized absorbance and emission spectra of aqueous dispersions of these differently sized PSP loaded with Nile Red and Itrybe are displayed in the SI in Fig. S5. Due to the dye staining of the particles via a previously optimized swelling procedure assessed, e.g., microscopy, the dye molecules can be assumed to be homogeneously distributed within the particles, and the spherical shape of the particles is preserved in the process.^12,41,42,44^ This allows for a data analysis based on Mie Theory, like with the unstained particles discussed above. The absorbance and emission spectra of Itrybe-loaded PSP are broadened compared to the parent molecule in ethanol, yet the respective absorption and emission maxima match. In the case of solvatochromic Nile Red, the absorbance and emission bands of Nile Red-stained PSP are red shifted with decreasing PSP size, pointing to an increasingly polar microenvironment faced by the dye molecules with reduced PSP size and hence increased surface-to-volume ratio. The corresponding ${\Phi }_{\mathrm{pl}}$ of aqueous dispersions of Nile Red- and Itrybe-loaded PSPs in dependence on PSP size and loading concentration, i.e., the average dye-dye distance are shown in Fig. 4. This figure reveals opposite trends for the particle size- and dye loading-dependent ${\Phi }_{\mathrm{pl}}$ of both dyes. As shown in the left panel of Fig. 4, the ${\Phi }_{\mathrm{pl}}$ values of Nile Red-stained PSP decreased with decreasing PSP size and increased surface-to-volume ratio. In contrast, for PSP containing Itrybe molecules, ${\Phi }_{\mathrm{pl}}$ decreased with increasing particle size (right panel of Fig. 4. For both dyes, an increase in dye loading concentration resulted in a diminution of ${\Phi }_{\mathrm{pl}}$ (see also SI, Fig. S7). We attribute these trends to (1) the influence of the polar particle environment particularly in the case of the charge transfer dye Nile Red, the fluorescence of which is known to be quenched by hydrogen bonding interactions and polarity as follows also from the solvatochromic emission behavior of Nile Red (see SI, Fig. S5), (2) dye-dye interactions, and (3) energy transfer processes between dye molecules in the PSP. The latter two factors are supported by the dependence of ${\Phi }_{\mathrm{pl}}$ on dye loading concentration (see Fig. 4 and SI, Fig. S7)^66–70^.* µ*_a_(λ) and *µ*_s_(λ) of the dispersed 1 µm and 100 nm dye loaded PSPs determined by radiation transport and Mie Theory are shown in Fig. 5. For the 25 nm sized particles the scattering contributions were too small to be used for quantification and represent the physical size limit for our study and method development. For the fit of *µ*_a_(λ) and *µ*_s_(λ), we considered the contribution of the dye molecules to the dielectric function of the particle matrix by increasing the imaginary part of the refractive index. In a first step, *n*_2_ was used as a free variable and it was assumed that the particle matrix is homogeneously loaded with dye molecules. The resulting σ_a_(λ) values are shown in Fig. 6. The resulting *N*_p_ and *r*_p_ values were also used for the determination of σ_a_(λ) from µ_a_(λ) calculated from the radiation transport theory. For the particle sizes and dye concentrations used here, the direct determination of σ_a_(λ) from Mie calculations and calculations using radiation transport theory are in good agreement as displayed in Fig. 5.

So far, we treated the real part of the refractive index and the imaginary part as independent quantities. In the Mie scattering calculations, we set the imaginary part of the dye-stained PSP according to an absorption line of the dye Nile Red or Itrybe but assumed that the real part equals that of unstained PSP. When Eqs. (16)–(18) are applied to the stained particles, a dye-concentration-dependent contribution to ${n}_{1}\left(\uplambda \right)$ that features an anomalous dispersion (i.e., locally, with the refractive index decreasing with wavelength) which was neglected so far and “Materials and methods” section for more details. We repeated the Mie scattering calculations including also the term due to the KK relations for the complex refractive index of the particles. Results of this physically more complete model are shown in the SI in Fig. S6 for 1 µm-sized PSP stained with Nile Red. Generally, ${\mu }_{a}\left(\uplambda \right)$ remains practically unaffected by the KK contribution to ${n}_{1}\left(\uplambda \right)$, whereas some spectral features of ${\mu }_{s}\left(\uplambda \right)$ changed noticeably. Since ${\mu }_{s}\left(\uplambda \right)$ is utilized to determine the particle size and particle number concentration, an error is introduced into this approach by omitting the KK term. For the 1 µm particles, we estimated the corresponding uncertainty to be about 5% for the mean particle diameter, which is accordingly underestimated, and to about 10% for the particle concentration, that is hence overestimated, depending on the exact workflow of the data analysis. For smaller particles, the effect of the KK relations on ${\mu }_{\mathrm{s}}\left(\uplambda \right)$ decreases and can be neglected for diameters ≤ 100 nm.

As shown in the left panel of Fig. 6, *σ*_a_(λ) linearly increased up to 7 × 10^4^ and 1.8 × 10^7^ incorporated Nile Red or Itrybe molecules for 100 nm and 1 µm PSP. The impact of dye loading on the effective particle properties, i.e., *Q*_abs_ and *n*_2_ is displayed in the right panel of Fig. 6. For 100 nm-sized PSP, *Q*_abs_ is considerably smaller compared to 1 µm PSP. An extrapolation shows that also for higher dye loading concentrations, i.e., *n*_2_, the efficiency increases linearly. For 1 µm particles the relation between *Q*_abs_ and *n*_2_ is no longer linear for dye concentrations exceeding the concentrations used in this study.

**Figure 7:**
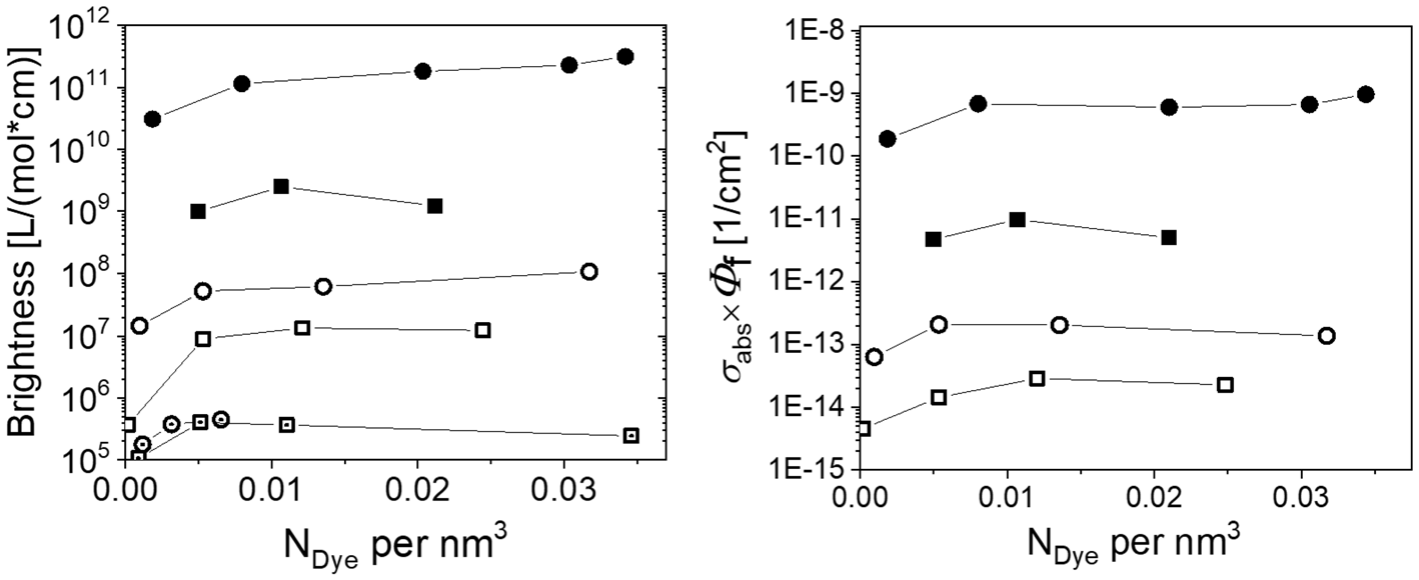
Left: Brightness with a unit of L/(mol*cm) calculated as the product of the number of dye molecules inside a particle (*N*_Dye_), the quantum yield and the molar decadic absorption coefficient of the respective dye. Right: Determined brightness values with a unit of (1/cm^2^) in dependence of the dye amount per nm^3^. 1 µm (full symbols) and 100 nm (open symbols) and 25 nm (open symbols with centered dot) PSP loaded with Nile Red (circles) and Itrybe (squares). Both graphs cover 7 orders of magnitude.

The product of ${\Phi }_{\mathrm{pl}}$ and *σ*_a_(λ) can be used to calculate the brightness (*B)* of a particle as done before for the *B* value of a dye molecule as shown in Fig. 7. For molecular luminophores like dye molecules, *B* is typically given as the product of ${\Phi }_{\mathrm{pl}}$ and the molar decadic absorption coefficient (ε(λ)) which is related to the oscillator strength of the emitting dipole. In contrast to dye molecules, *σ*_*a*_(λ) of particles scale with *r*_p_, *n*_2_, and ${\Phi }_{\mathrm{pl}}$. The latter is related to the number of dye molecules incorporated into the particle (see Figs. 4 and  6), dye-dye interactions, and dye-specific properties like the oscillator strength and ${\Phi }_{\mathrm{pl}}$ in the respective environment. Earlier, we calculated the particle brightness from the product of the number of molecules per particle, the corresponding *ε *of the dye determined in a model solvent such as BOB, and the measured ${\Phi }_{\mathrm{pl}}$ of the particle dispersion (${{N}_{\mathrm{Dye}}\cdot\upvarepsilon ({\lambda }_{ex})\cdot \Phi }_{\mathrm{pl}}$)^27,71^, see Fig. 7 (left panel). This approximation, that is valid for small particles and low dye loading concentrations, has several drawbacks. As shown in the SI in Fig. S5, (1) ε can depend on dye environment and can only be measured in a solvent which mimics the dye environment inside the particle. Mimicking implies here ideally a comparable polarity and refractive index. (2) For the determination of *N*_Dye_, the particles need to be dissolved and (3) for high dye loading concentrations, the *σ*_*a*_(λ) must not necessarily be linearly related with the amount of incorporated dye molecules. An advantage of this method is that *B* values of very small particles like. 25 nm-sized polymer beads can be determined (Fig. 7, left panel).

The definition of particle brightness by Eq. (19) clearly represents a more general approach as all parameters are experimentally accessible with integrating sphere spectroscopy (see Fig. 7, right panel). A direct comparison of the two approaches utilized for the determination of B values of luminescent particles in units of (L/(mol*cm) and (1/cm^2^) is shown in Fig. 7 (and SI, Fig. S8, for one swelling batch).

19 $$B={\sigma }_{a}{\Phi }_{pl}$$

As can be seen in Figs. 4, 6, and 7 and in Supplementary Fig. S8, apparently ${\Phi }_{\mathrm{pl}}$ is not the quantity, the value of which dominates the measured signal amplitude recorded for a luminescent particle, but the *σ*_*a*_(λ). For example, the latter quantity is a factor of 100 to 1000 larger for 1 µm PSP compared to 100 nm PSP. 1 µm sized particles loaded with Itrybe have a ${\Phi }_{\mathrm{pl}}$ of less than 1% but show a *B* value higher than that of 100 nm sized PSP loaded with Nile Red that exhibit a ${\Phi }_{\mathrm{pl}}$ of 76%. Figure 7 indicates optimum dye loading concentrations for high *B* values. Apparently, for both dyes, the highest *B* values were obtained for a dye loading concentration range of 0.83 × 10^–8^ to 2.1 × 10^–8^ mol per mg PSP.

Finally, to cancel out the influence of the physical size of the luminescent particles and to introduce a size-independent luminescence efficiency (*LE*) for luminescent particles, we calculated the luminescence efficiency (*LE*) of dispersions of luminescent particles as the product of the photoluminescence quantum yield ${\Phi }_{\mathrm{pl}}$ and the absorption efficiency (*Q*_abs_) of a particle:

20 $$LE={Q}_{abs}{\Phi }_{\mathrm{pl}}$$

**Figure 8:**
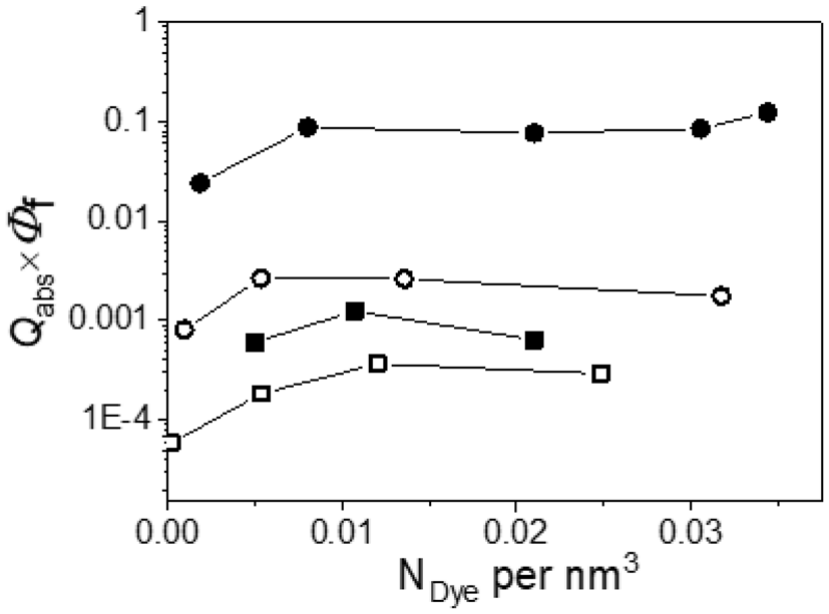
Luminescence efficiency *LE* of 1 µm (full symbols) and 100 nm (open symbols) PSP loaded with Nile Red (circles) and Itrybe (squares).

*LE* values as defined here by us is a unitless number between 0 and 1 and represents the ratio of the photons incident on the particle cross section and the photons emitted by the particle (see Fig. 8).

In terms of *LE* (Fig. 8), 100 nm Nile Red-stained PSP clearly exceed 1 µm Itrybe-loaded PSP. In addition, the particle *LE* scaled over the incorporated dye amount per 3 mg PSP is shown in the SI in Fig. S9. For applications where particle ensembles are utilized and not single particles like, e.g., analyte-responsive nanosensors, carrier beads for bead-based assays or light or energy conversion layers, particle *LE* reflecting the photon shifting efficiency may be the more important parameter for the characterization of luminescent particles and the comparison of their performance.

### Conclusion and outlook

In summary, we presented a brightness (*B*) scale for the performance evaluation and comparison of light-scattering dispersions of luminescent particles that expresses the signal-relevant optical properties of the particles in terms of fundamental spectroscopic quantities. This brightness scale is based upon measurements of the absorption cross section and the photoluminescence quantum yield (*Φ*_pl_) with a single integrating sphere setup custom-designed to enable the absolute determination of *Φ*_pl_ and transmittance and diffuse reflectance measurements.

This concept of classifying particle *B* values, was exemplarily shown for aqueous dispersions of quasi-monodisperse 25 nm-, 100 nm-, and 1 µm-sized spherical polystyrene particles, homogeneously core stained with two dyes in varying concentrations. These fluorophores were chosen to differ in their solvatochromic behavior and spectral overlap between their absorption and emission bands and hence reabsorption. Subsequently, *Φ*_pl_ of these materials and the optical properties, i.e., the absorption and scattering coefficients µ_a_ and µ_s_ were determined from solutions of the radiation transport theory based on reflection and transmission measurements of the particle dispersions with our integrating sphere setup. This procedure was validated with unstained materials. For our model compounds, we further calculated *σ*_*a*_(λ), *n*_2_, and *Q*_abs_(*λ*) in dependence of the number of incorporated dye molecules taking into account Mie theory. Finally, we defined a unitless luminescence efficiency (*LE*) as the product of the absorption efficiency and *Φ*_pl_ which effectively represents the fraction of absorbed and reemitted photons of a particle and determined these values for dye-stained particles.

Overall, the described procedure allows for the determination of scattering and absorption properties of light scattering particle-containing media, the determination of number concentrations, and material constants as well as the identification of the particles with the best luminescence performance for technical and biological applications with just one integrating sphere setup. The lower limit for particle size is around 50 nm, depending on the particle and solvent refractive index. This concept, that can be also realized with a photometer equipped with an integrating sphere and a stand-alone integrating sphere setup for the absolute measurement of *Φ*_pl_ values, has the potential to overcome relative intensity comparisons and can provide a more general intensity scale for photoluminescent light scattering particles. In the future, we will further explore the implementation of the Kramers Kronig relations into the Mie fitting algorithm regarding an optimized parameter weight.

## Supplementary Information

Supplementary Figures.

## Data Availability

All data generated or analyzed during this study are included in this published article (and its Supplementary Information (SI) files) or are available upon request from the corresponding authors.
