# Supplementary material for: Efficiency scale for scattering luminescent particles linked to fundamental and measurable spectroscopic properties
Source: Sci Rep. 2023 Apr 17;13:6254. doi: 10.1038/s41598-023-32933-6 (PMC10110600; doi:10.1038/s41598-023-32933-6)
Supplement: Supplementary file 1 — Supplementary Figures. [file 41598_2023_32933_MOESM1_ESM.pdf]

Efficiency scale for scattering luminescent particles linked to fundamental and measurable spectroscopic properties

Supporting information

Christian Würth[1], Thomas Behnke[1], Jonas Gienger[2], Ute Resch-Genger[1]

[1] Division *Biophotonics*, Bundesanstalt für Materialforschung und –prüfung, Richard-Willstaetter Str. 11, 12489 Berlin, Germany

[2] Physikalisch-Technische Bundesanstalt (PTB), Abbestr. 2–12, 10587 Berlin, Germany

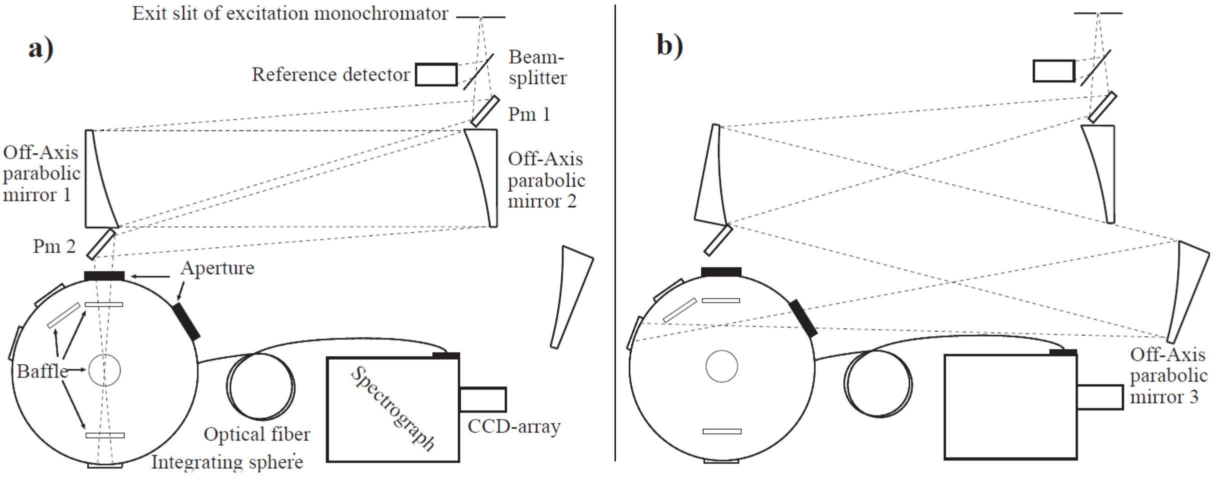

Figure S1: Schematic drawing of the integrating sphere setup. Pm stands for plane mirror.

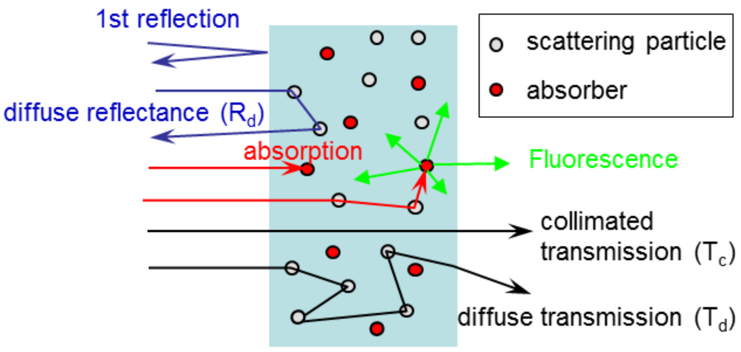

Figure S2: Illustration of the light propagation in strongly scattering and luminescent media.

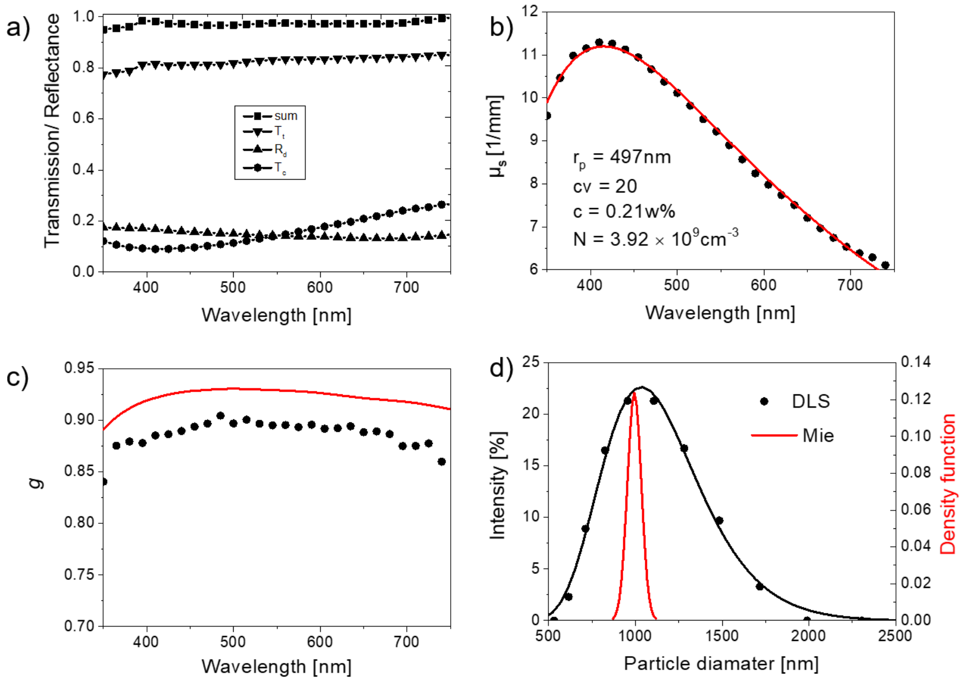

Figure S3: Dispersion of polystyrene particles (PSP) with d~1 µm: a) Diffuse reflectance (T_d, triangles), total (T_t, triangles) and collimated transmission (T_c, solid circles) and sum of R_d and T_t (solid squares). b) Scattering coefficient (μ_s) calculated from the radiation transport theory (solid squares) and Mie theory (red line). c) Anisotropy factor *g* calculated from the radiation transport theory (solid squares) and Mie theory (red line). d) Size distribution function determined with DLS (solid circles, black line, d_{DLS} = 990 nm) and Mie theory (red line).

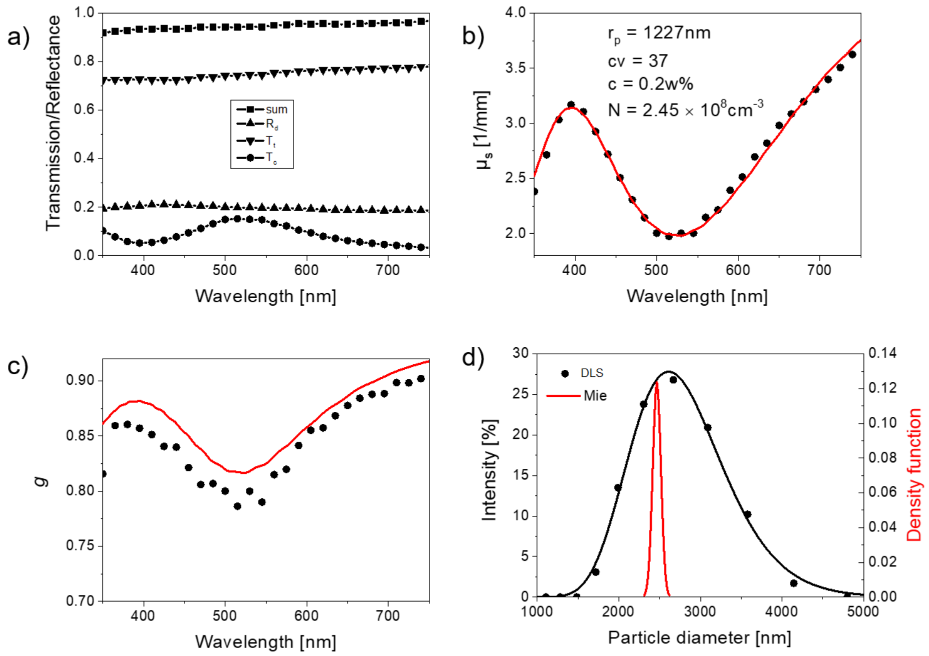

Figure S4: Optical properties of dispersion of PSP with d~2.5 µm: a) Diffuse reflectance (T_d, triangles), total (T_t, triangles) and collimated transmission (T_c, solid circles) and sum of R_d and T_t (solid squares). b) Scattering coefficient (μ_s) calculated from the radiation transport theory (solid squares) and Mie theory (red line). c) Anisotropy factor *g* calculated from the radiation transport theory (solid squares) and Mie theory (red line). d) Size distribution function determined with DLS (solid circles, black line, d_{DLS} = 2592 nm) and Mie theory (red line).

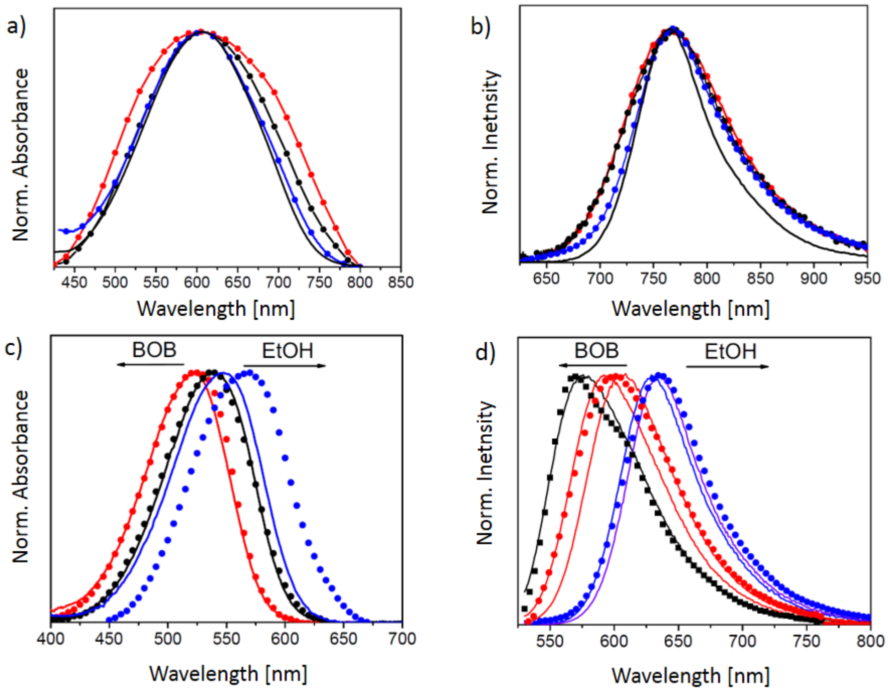

Figure S5: Nomalized absorbance (left) und normalized emission (right) spectra of Itrybe (top) and Nile Red (bottom) incorporated into 1 µm PSP (red circles), 100 nm PSP (black circles), and 25 nm PSP (blue circles) and corresponding spectra of the free dyes in solution (lines). Itrybe was dissolved in EtOH, Nile Red in mixtures of BOB/EtOH of varying BOB-to-EtOH ratio. c) 90 % / 10 % (red lines); 50 % / 50 % (black lines) und pure EtOH (blue lines). d) 99 % / 1 % (red lines); 96 % / 4 %, 90 % / 10 % (black lines); 30 % / 70 %, 0 % / 100 % (blue lines).[1] BOB acts here as model system for the PS matrix. Itrybe is not soluble in BOB.

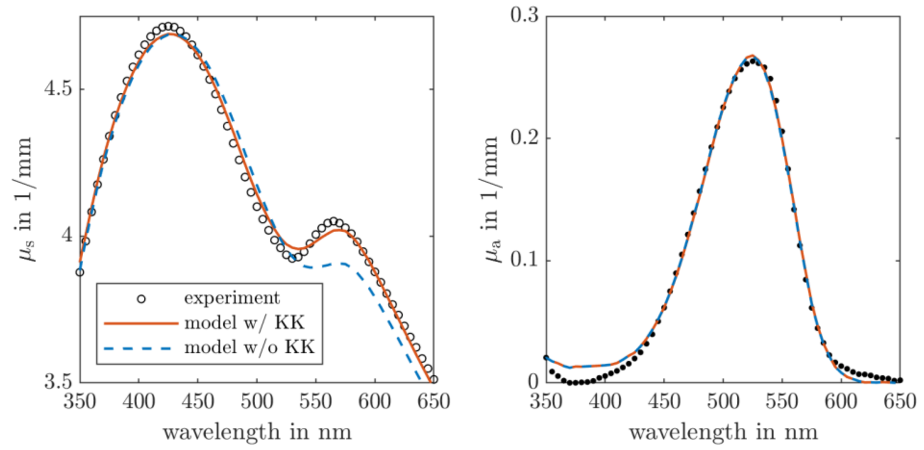

Figure S6: Effect of Kramers-Kronig (KK) relations on Mie scattering simulations of μ_s and μ_a of 1µm PSP, stained with 1.5mM NR. Red solid lines: simulations taking into account the contribution of the absorption spectrum to the real part of the RI [Eqs. (16), (18) in the main text] and blue dashed lines: omitting it. Model parameters (size distribution, particle concentration, BOB/EtOH ratio of absorption spectrum in Fig. S5) were obtained from a simultaneous fit of $\mu_s(\lambda)$ and $\mu_a(\lambda)$ with the KK term enabled and are identical in both cases.

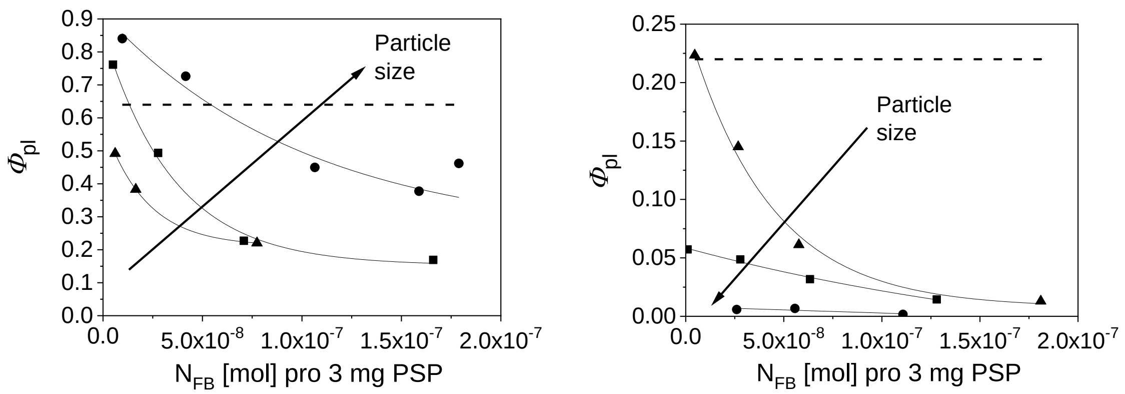

Figure S7: Photoluminescence quantum yields (Φ_{pl}) of 1 µm (circles), 100 nm (squares), and 25 nm (triangles) PSP loaded with Nile Red[2] (left) and Itrybe (right) in dependence of the incorporated amount of dye molecules per 3 mg PSP. The solid lines are only a guide to the eyes. The dashed lines indicate the Φ_{pl} of the free dye in EtOH.

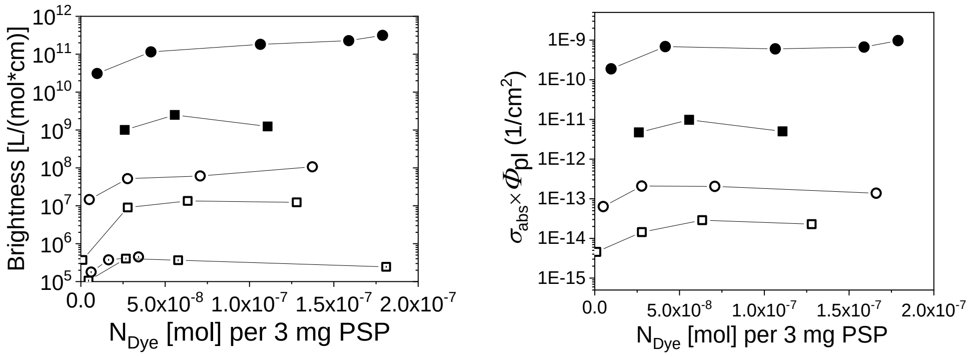

Figure S8: Left: Brightness with a unit of (L/(mol*cm) calculated as the product of the number of dye molecules inside a particle (N_{Dye}), Φ_{pl} , and the molar decadic absorption coefficient of the respective dye. Right: Determined brightness values with a unit of (L/mol*cm) in dependence of the dye amount incorporated into 3 mg PSP (one swelling batch). 1 µm (full symbols) and 100 nm (open symbols) and 25nm (open symbols with centered dot) PSP loaded with Nile Red (circles) and Itrybe (squares). Both graphs cover 7 orders of magnitude.

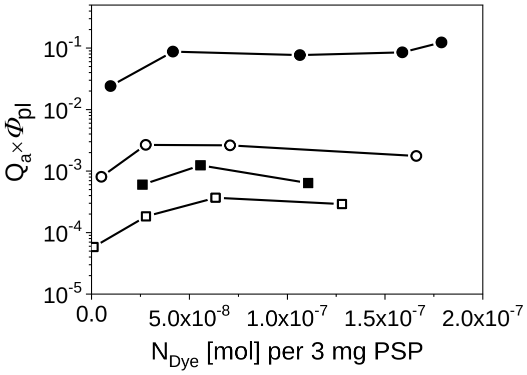

Figure S9: Unit-less particle efficiency value calculated from the absorbance efficiency and Φ_{pl} in dependence of the incorporated dye amount in 3 mg PSP (one swelling batch). 1 µm (full symbols) and 100 nm (open symbols) PSP loaded with Nile Red (circles) and Itrybe (squares).

Literatur

1 Behnke, T. *et al.* Encapsulation of Hydrophobic Dyes in Polystyrene Micro- and Nanoparticles via Swelling Procedures. *Journal of Fluorescence* **21**, 937-944, doi:10.1007/s10895-010-0632-2 (2011).

2 Behnke, T. *et al.* Target-specific nanoparticles containing a broad band emissive NIR dye for the sensitive detection and characterization of tumor development. *Biomaterials* **34**, 160-170, doi:10.1016/j.biomaterials.2012.09.028 (2013).
